# Experience with cochlear implants in Greenlanders with profound hearing loss living in Greenland

**DOI:** 10.3402/ijch.v72i0.20974

**Published:** 2013-08-05

**Authors:** Preben Homøe, Ture Andersen, Aksel Grøntved, Lone Percy-Smith, Michael Bille

**Affiliations:** 1 Department of Otolaryngology, Head and Neck Surgery and Audiology, Rigshospitalet, University of Copenhagen, Copenhagen, Denmark; 2 Department of Audiology, Odense University Hospital, Odense, Denmark; 3 Department of Otolaryngology, Head and Neck Surgery, Odense University Hospital, Odense, Denmark

**Keywords:** cochlear implant, hearing, deafness, Inuit, Greenland

## Abstract

**Objective:**

Cochlear implant (CI) treatment was introduced to the world in the 1980s and has become a routine treatment for congenital or acquired severe-to-profound hearing loss. CI treatment requires access to a highly skilled team of ear, nose and throat specialists, audiologists and speech-language pathologists for evaluation, surgery and rehabilitation. In particular, children treated with CI are in need of long-term post-operative auditory training and other follow-up support.

**Design:**

The study is retrospective with updated information on present performance.

**Results:**

Since 2001, a total of 11 Greenlandic patients living in Greenland have been treated with CI, 7 children and 4 adults. Of these children, 4 use oral communication only and are full-time CI-users, 2 with full-time use of CI are still in progress with use of oral communication, and 1 has not acquired oral language yet, but has started auditory and speech training. Six children attend mainstream public school while one child is in kindergarten. Of the adults, only 1 has achieved good speech perception with full-time use of CI while 3 do not use the CI.

**Discussion:**

From an epidemiological point of view, approximately 1–3 children below 6 years are in need of a CI every second year in Greenland often due to sequelae from meningitis, which may cause postinfectious deafness. Screening of new-borns for hearing has been started in Greenland establishing the basis for early diagnosis of congenital hearing impairment and subsequent intervention. The logistics and lack of availability of speech therapists in Greenland hampers possibilities for optimal language and speech therapy of CI patients in Greenland. This study aims at describing the results of CI treatment in Greenlanders and the outcome of the CI operations along with the auditory and speech/language outcomes. Finally, we present a suggestion for the future CI treatment and recommendations for an increased effort in the treatment and rehabilitation of implanted patients in Greenland.

Persons with a profound hearing impairment, either congenital or acquired or pre- or post-lingual, in the remote Arctic areas are often left alone without much rehabilitation or support from the authorities. In Greenland, there used to be a school for the deaf but it has since been closed down. Since introduction in the 1980s, cochlear implantation (CI) has become a routine treatment option in patients with a severe-to-profound hearing impairment and limited benefit from conventional hearing aids. A CI consists of an electrode array implanted in the cochlea and connected to a receiver placed under the skin. The external microphone and sound processor transmit signals to the internal device providing electrical stimulation to the auditory nerve that is conveyed to the brain and perceived as sound. Thus, it has become possible to restore speech comprehension ability in postlingually deafened patients. Children with a congenital severe-to-profound hearing impairment are, with the use of CI, able to develop speech perception and spoken language skills comparable to normal hearing children when implanted early, preferably before 12 months of age (1, 2). It has been shown that quality of life after CI can be improved substantially (3). Also, CI has been shown to be cost-effective in industrialised countries but with varying results between studies (4). Cost-effectiveness of CI in Greenland or remote areas has not been examined. A candidate for CI in Greenland is sent to Denmark for evaluation and surgery. Post-operative rehabilitation is supposed to take place in Greenland with some guidance from the speech language pathologist from the CI centre. We have examined the performance and status of the implanted Greenlandic patients who live in Greenland.

The study is retrospective and covers the period between 2001 and 2011. The medical records have been scrutinised, and the districts where the patients live have been contacted for information on language, school attendance and the use of the CI.

## Results

The first Greenlandic patient received a CI in 2001. During the 9 years, 7 children aged between 2 and 5 years and 4 adults aged between 42 and 50 years have had CI done. The gender distribution for the children was 3 girls and 4 boys and for the adults, 2 females and 2 males. Of the children, 2 had hearing impairment due to pneumococcal meningitis, 4 had non-specified congenital hearing loss and 1 was congenitally deaf due to genetically verified Connexin 26 related hearing impairment, 35delG mutation (GJB2). Of the adults, 3 were profoundly hearing impaired due to pneumococcal meningitis and 1 was impaired after treatment with aminoglycoside. Six of the 7 children were implanted bilaterally, 2 simultaneous and 4 sequential, while 3 of the 4 adults were implanted unilaterally. Nine patients were implanted with Nucleus CI24RE CA electrodes and 2 adult patients were implanted with split-electrodes due to ossification of the cochlea after pneumococcal meningitis. The 2 patients (aged 49 and 50 years) with cochlear ossification were implanted 4 months and 3.5 years after the meningitis episode, respectively, while the one (46 years-old) without ossification was implanted 5 months after the meningitis episode. The 2 children (aged 27 and 39 months) with hearing loss after pneumococcal meningitis were implanted 1.6 and 2.1 years after the infection, respectively. The median age of identification for the five congenitally hearing impaired children was 1.5 years (range 0.8–3.7) and the median age of implantation was 2.9 years (range 2.1–5.7 years). Four patients live in Nuuk, 3 in Tasiilaq, 1 in Ilulissat, 1 in Sisimiut, 1 in Nanortalik and 1 in Kangaatsiaq. Two patients experienced a post-operative infection and were treated with antibiotics. However, one of these patients needed the CI to be removed and a later reimplantation to control the infection.

One patient suffered from post-implantation facial nerve stimulation and needed switch-off of the offending electrodes. Contact to the local districts in Greenland and one of the two speech language therapists in Greenland revealed that 4 children use oral communication mode and are full-time CI-users, 2 are full-time users of CI and are still in progress using oral communication and 1 has not developed spoken language as yet but has started auditory and speech training. One of the children is also psychomotorically retarded. Six children attend mainstream public school while 1 is in mainstream kindergarten. Of the adults, only 1 (deafened after exposure to gentamycin) has developed speech comprehension with full-time use of CI. The other 3 adults who were deafened after meningitis and who have additional alcohol problems do not use the CI and are left with lip-reading.

## Discussion

It is expected that between 1 and 3 children are either born deaf or become deaf or attain profound hearing loss mainly after meningitis in Greenland every year. Most deafness in Greenland relates to infections such as meningitis, which is potentially preventable. CI is one of the major breakthroughs in the world of medicine. The technology offers the possibility to establish or re-establish hearing in otherwise totally deaf or profoundly hearing impaired persons. In the case of a severe-to-profound hearing impairment with only limited benefit of conventional hearing aids, CI enables speech comprehension and spoken language communication (5). Early identification and intervention with the fitting of hearing aids before 6 month of age is important for children with a congenital or early-acquired hearing impairment in order to achieve optimal results in terms of spoken language and speech development (6). When CI is needed, implantation is preferred before children reach 12 months of age (2).

This study demonstrates delayed identification of children with severe or profound congenital hearing impairment typical for a population without a neonatal hearing-screening programme (7). However, a significant delay from identification to effective treatment is also shown. Universal hearing screening in new-borns was introduced in Greenland in 2007 as a 2-stage screening test with transient evoked otoacoustic emissions. In case of failure of the screening procedure, the new-born is referred for further examination and audiological testing by a visiting audiological physician at the local hospital or in the ear, nose and throat department at the main hospital in Nuuk. Establishing neonatal hearing screening is essential for early detection and intervention but emphasis must still be placed on immediate audiological evaluation when screening is not passed and timely referral for specialised evaluation in Denmark whenever needed. Timely identification of post-meningitis bilateral severe-to-profound hearing loss and prompt referral for definitive audiological evaluation and appropriate treatment with CI is required due to the risk of intracochlear fibrosis or ossification precluding proper electrode insertion (8). This study shows a significant delay for all the post-meningitis patients leading to suboptimal electrode insertion in 2 cases. Therefore, physicians in Greenland should show alertness of hearing loss after meningitis cases, and all meningitis patients in Greenland should have performed a hearing test as soon as possible after the incident. If there is any suspicion of profound hearing loss, the patient should be discussed immediately with the audiological specialists in Denmark. A problem in Greenland is the limited access to auditory and verbal training as there are only 2 trained speech therapists in Greenland to take care of the entire population of 56,000, including speech-related problems in school children. Also, education and support to the parents of implanted children are crucial to achieve the most optimal speech and language results (1). In Greenland, the important post-operative follow-up and treatment is in need of further attention in order to increase the outcome of treatment with CI. A rehabilitation programme would enhance this, and it is suggested that this be developed as soon as possible. This could be done by including the group of dedicated health care workers involved in this field, such as physicians in audiology, speech therapists, otosurgeons, the local district physicians and the social and child care authorities in the local districts. A programme could involve the use of the Internet such as Skype or other telemedicine facilities, which are already established and working in Greenland. A yearly follow-up by Danish specialists may be suggested in order to attend to potential problems as early as possible. Language barrier may also be an obstacle. It is therefore important that Greenland has enough updated speech therapists who can speak the Greenlandic language. This is increasingly important as the school for the deaf has been closed in Greenland. Other existing handicaps or disabilities in some of the affected patients or social deprivation could also interfere with the outcome of cochlear implantation. An important aspect of success after CI is the cooperation and attitude of the patients and their families. The selection of the patients is therefore of major clinical significance and should be performed meticulously. This has now resulted in a decision by the authorities in Greenland not to continue with CI in adults but only offer this treatment to children.

## Conclusions

CI treatment is difficult to perform optimally in Greenland. CI treatment of severe or profoundly hearing impaired Greenlandic children can be performed successfully. Earlier intervention in both congenital and acquired profound hearing loss is needed. Skype or telemedicine could be a valuable tool in the rehabilitation process. Language barrier and social problems are obstacles. There is a need for systematic auditory and verbal rehabilitation. A yearly control and follow-up by a CI specialised speech-language pathologist is suggested. A program for rehabilitation of all Greenlandic CI patients is urgently needed.
